# Socioeconomic stratification in the association between tea consumption and skeletal muscle mass among oilfield workers

**DOI:** 10.3389/fnut.2026.1777775

**Published:** 2026-04-24

**Authors:** Haobiao Liu, Yingjie Cai, Jing Tang, Pei Li, Licheng Yang, Xuefeng Yu, Qingsong Li, Abebe Feyissa Amhare, Ziwei Guo, Zhiyong Du, Jinsong Li, Jing Han

**Affiliations:** 1 Ningxia Gem Flower Hospital, Yinchuan, Ningxia, China; 2 School of Public Health, Health Science Center, Xi’an Jiaotong University, Xi’an, Shaanxi, China; 3 Department of Comprehensive Orthopedics, The Second Affiliated Hospital of Xi’an Jiaotong University, Xi’an, Shaanxi, China; 4 Xi’an Gem Flower Changqing Hospital, Xi’an, Shaanxi, China

**Keywords:** occupational population, oilfield workers, skeletal muscle mass, socioeconomic status, tea consumption

## Abstract

**Objectives:**

Tea consumption is a culturally embedded behavior in China, but its association with skeletal muscle health remains unclear, particularly in occupational populations. This study examined the relationship between tea consumption and appendicular skeletal muscle mass (ASM) among oilfield workers and assessed whether socioeconomic status (SES) modifies this association.

**Methods:**

Multivariable linear regression models were used to evaluate the association between tea consumption and ASM. Smooth curve fitting analyses were conducted to visualize potential dose-response relationships. SES-stratified and interaction analyses were performed to examine effect modification. Sensitivity analyses were carried out to test the robustness of the findings.

**Results:**

In fully adjusted models, low- and high-level tea intake were associated with 0.105 and 0.137 unit increases in ASM in the overall population, respectively. SES-stratified analyses showed that only high-level tea intake was significant in the low SES group, whereas in the high SES group, both low- and high-level intake were positively associated with ASM. No significant associations were observed in the medium SES group. Smooth curve fitting further supported these SES-specific patterns observed in linear regression. Interaction analysis indicated that the association between low-level tea consumption and ASM was stronger among individuals with high SES (*P* for interaction = 0.031). Sensitivity analyses yielded consistent findings.

**Conclusion:**

Tea consumption is positively associated with skeletal muscle mass among oilfield workers, with the strength of this association varying across SES. These findings highlight the importance of considering social context when promoting dietary strategies to support muscle health in occupational populations.

## 1 Introduction

Health inequalities across socioeconomic strata remain a persistent global challenge. Socioeconomic status (SES) profoundly shapes individuals’ exposure to health risks and access to protective resources (1, 2). A well-established “social gradient in health” indicates that individuals with higher SES generally experience better physical function and lower morbidity than those with lower SES (1), even within relatively homogeneous occupational groups. However, the magnitude and direction of health behavior benefits often differ by SES, leading to complex patterns of social inequality in health outcomes.

Lifestyle behaviors related to diet and daily routines are widely regarded as modifiable determinants of chronic disease and functional decline. Regular physical activity, balanced dietary patterns, and other health-related behaviors have been associated with reduced risks of cardiometabolic diseases, biological aging, and psychiatric disorders (3–6). However, accumulating evidence suggests that the health returns of these behaviors are not socially uniform. The magnitude—and in some cases the direction—of behavioral benefits may differ substantially across socioeconomic groups (7–9). For instance, previous research has shown that the mortality risk reduction associated with physical activity tends to be more pronounced among individuals with lower SES, potentially due to their higher baseline risk and greater room for improvement (8). In contrast, moderate alcohol intake has been reported to exhibit a stronger protective association with ischemic heart disease mortality in higher-SES groups than in lower-SES groups, whereby the same level of alcohol consumption yields more adverse consequences for disadvantaged populations (9). These observations highlight the importance of situating dietary and lifestyle behaviors within their broader social and structural contexts.

Tea consumption is a culturally embedded dietary behavior and one of the most widely consumed beverages worldwide. In East Asian societies, tea drinking is closely linked to traditional health beliefs, social norms, and daily routines. Experimental and epidemiological studies suggest that tea contains bioactive compounds—particularly polyphenols such as catechins—that exert antioxidant, anti-inflammatory, and metabolic regulatory effects potentially relevant to muscle maintenance (10–12). Mechanistically, catechins, including epigallocatechin gallate, have been shown to modulate oxidative stress and inflammatory signaling pathways involved in muscle protein turnover, mitochondrial function, and age-related muscle atrophy (13, 14). In addition, L-theanine, a tea-specific amino acid, may influence neuroendocrine stress responses and immune regulation, thereby indirectly affecting muscle metabolism under sustained physiological or occupational stress (15). Although the bioavailability and effective dosage of these compounds vary by tea type and preparation, habitual tea consumption has been proposed as a meaningful population-level source of these bioactives. Despite these biological plausibility considerations, population-based evidence linking tea consumption to musculoskeletal health remains limited and inconsistent, with studies reporting both favorable and null associations (16, 17). This heterogeneity suggests that the health effects of tea may depend not only on intake level but also on broader social and behavioral contexts, including socioeconomic conditions.

Skeletal muscle mass is a key component of metabolic health and physical function and plays a critical role in preventing frailty, disability, and chronic disease in adulthood. Declines in muscle mass are associated with increased risks of morbidity, reduced work capacity, and adverse aging outcomes. Previous studies have documented marked socioeconomic disparities in muscle strength and physical performance, with individuals of lower SES more likely to experience muscle-related impairments due to factors including nutritional inadequacy, higher physical workload, and limited access to preventive health resources (18, 19). Despite this, little is known about whether common dietary behaviors—such as tea consumption—may mitigate muscle loss, or whether their potential benefits differ across socioeconomic strata.

Occupational populations provide a valuable context for examining these questions. Oilfield workers, in particular, are exposed to distinctive physical demands and environmental stressors, while often sharing relatively standardized employment structures and healthcare access. This setting offers an opportunity to disentangle the role of socioeconomic differences from broader occupational factors. Understanding whether the association between tea consumption and skeletal muscle mass varies by SES in such populations may yield important insights into how culturally embedded eating behaviors intersect with social inequality to influence physical health.

In this study, we examined the association between tea consumption and skeletal muscle mass among Chinese oilfield workers, with a specific focus on socioeconomic disparities. We hypothesized that the relationship between tea drinking and muscle mass would vary across SES levels, reflecting differential physiological and contextual returns of the same dietary behavior. By adopting a social epidemiological perspective within a nutritional framework, this study aims to contribute evidence on how daily eating behaviors may differentially shape chronic disease-related outcomes across social strata.

## 2 Materials and methods

### 2.1 Study population and design

This cross-sectional study was conducted among employees of a petroleum enterprise in Xi’an, Shaanxi Province, China. Participant recruitment took place at the enterprise-affiliated hospital between October and December 2022. During on-site health examination visits, trained investigators administered face-to-face questionnaires, and anthropometric and laboratory data were obtained from the hospital’s electronic medical record system. Eligible participants were required to be aged 18 years or older, have been employed for at least 1 year, and voluntarily agree to participate. Individuals were excluded if they were pregnant or breastfeeding, had severe neurological or psychiatric disorders that could impede completion of the survey.

**FIGURE 1:**
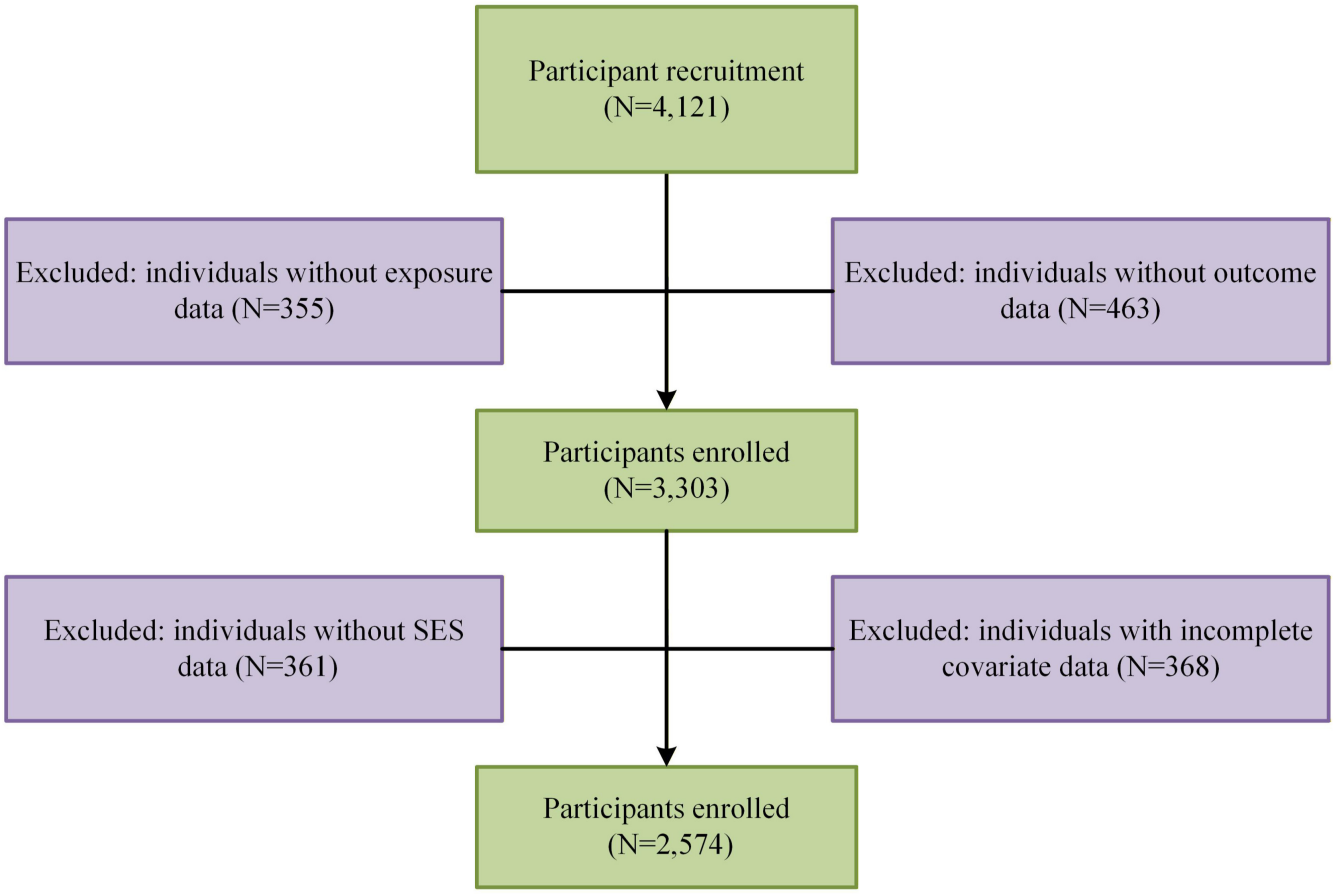
Flow diagram of participants included in this study.

A total of 4,121 employees were initially screened. Of these, 355 were excluded due to missing exposure variables, 463 due to missing outcome data, 361 due to missing SES data, and 368 due to incomplete covariate information. After applying all inclusion and exclusion criteria, 2,574 participants were included in the final analyses (Figure 1). All participants provided written informed consent prior to enrollment. The study protocol was reviewed and approved by the Medical Ethics Committee of Xi’an Jiaotong University, and all procedures adhered to the ethical principles of the Declaration of Helsinki.

### 2.2 Assessment of tea consumption

Habitual tea consumption was assessed using a structured, face-to-face questionnaire administered by trained study staff. Participants were first asked whether they consumed tea. For those reporting tea consumption, detailed information on frequency and typical daily quantity was collected. To minimize individual reporting bias, participants were provided with a standardized 250 mL cup as a reference for estimating tea intake. Based on these responses, participants were categorized as non-tea drinkers, low-consumption tea drinkers, and high-consumption tea drinkers, with the median intake among tea drinkers used to define low and high consumption. Detailed data on tea type, brewing strength, preparation methods, or chemical composition were not available and therefore not incorporated into the exposure definition.

### 2.3 Assessment of skeletal muscle mass

Appendicular skeletal muscle mass (ASM) was estimated using an anthropometric equation specifically developed and validated in Chinese adults (20). Standing height and body weight were obtained by trained technicians using a wall-mounted stadiometer and a calibrated digital scale. Age was recorded in years, and sex was coded as one for males and two for females. ASM was computed using the following formula:


A⁢S⁢M=0.193×b⁢o⁢d⁢y⁢w⁢e⁢i⁢g⁢h⁢t+0.107×h⁢e⁢i⁢g⁢h⁢t

−4.157×sex−0.037×age−2.631

The validity of this model has been confirmed against dual-energy X-ray absorptiometry (DXA), with the original validation study reporting an adjusted R^2^ of 0.90 and a standard error of estimate of 1.63 kg, demonstrating strong concordance and supporting its application in epidemiological research. After calculating ASM, skeletal muscle mass index was derived by dividing ASM by height squared (kg/m^2^), and this index was used as the primary outcome measure of muscle status in the present analysis.

### 2.4 Assessment of SES

Socioeconomic status was constructed using educational attainment and annual individual income, two indicators commonly used in social epidemiology to capture both long-term and current socioeconomic position. Educational attainment was included as a relatively stable marker of early-life and cumulative socioeconomic advantage, reflecting long-term access to knowledge, cognitive resources, and health-related decision-making capacity. Education was categorized according to standardized stages of the Chinese education system into six levels: primary school, lower secondary school, upper secondary school, post-secondary non-tertiary education, bachelor’s degree, and graduate degree. Annual income, including all sources of earnings and allowances, was coded into four ordered categories: ≤100,000 RMB, 110,000–150,000 RMB, 160,000–200,000 RMB, and >200,000 RMB. All participants were oilfield workers with comparable occupational roles and identical health insurance coverage, which minimized heterogeneity related to occupational grade and access to social security and allowed education and income to serve as the primary dimensions capturing socioeconomic variation within this occupational population. Both variables were standardized using z-scores and then averaged to generate a composite SES index, with higher values indicating higher socioeconomic status. The composite index was divided into tertiles to define low, middle, and high SES levels, reflecting socioeconomic gradients within the study population.

### 2.5 Covariates

Covariates were selected based on prior evidence and biological plausibility (21, 22). The following variables were included: age, sex, ethnicity, marital status, shift work, chemical exposure, noise exposure, dust exposure, cigarette smoking, alcohol consumption, physical activity, hypertension, diabetes, dyslipidemia, and cardiovascular disease. Definitions and categorizations of covariates are presented in Supplementary Table 1.

### 2.6 Statistical analysis

Descriptive statistics were presented by tea consumption level. Continuous variables were described as means ± standard deviations (SD), and categorical variables as counts and percentages. Group differences were evaluated using analysis of variance or chi-square tests.

Multivariable linear regression models were applied to estimate the association between tea consumption and the ASM index, with results reported as regression coefficients (β) and corresponding 95% confident intervals (CI). Three progressively adjusted models were developed. The crude model included no covariate adjustment. Model 1 adjusted for age, sex, ethnicity, and marital status. Model 2 additionally adjusted for shift work, exposure to chemical substances, noise and dust exposure, cigarette smoking, alcohol consumption, physical activity, hypertension, diabetes, dyslipidemia, and cardiovascular disease. To examine whether the association between tea consumption and the ASM index varied across SES strata, a multiplicative interaction term was added to the fully adjusted model.

To further visualize the dose-response relationship, smooth curve fitting analyses were additionally conducted. Because the raw tea consumption variable ranged widely, we rescaled the variable by dividing by 500 to reduce the influence of extreme values and improve model stability before fitting the spline model, with three knots placed at the 5th, 50th, and 95th percentiles.

Sensitivity analyses were also performed to evaluate the robustness of the findings, including using binary tea drinking status and applying multiple imputation for missing covariates. All analyses were conducted using R software (version 4.4.0) and EmpowerStats.^1^ Statistical significance was defined as *P* < 0.05.

## 3 Results

### 3.1 Baseline characteristics of participants

A total of 2,574 oilfield workers were included in the final analysis. The mean age of participants was 41.05 ± 8.21 years, and 66.08% were males. Baseline characteristics stratified by tea consumption level are summarized in Table 1. Clear sociodemographic and behavioral differences were observed across tea consumption groups. Compared with non-tea drinkers, individuals with higher tea intake tended to be older and were substantially more likely to be male (*P* < 0.001). Although ethnicity distribution was similar across groups, high-level tea drinkers showed higher proportions of regular physical activity and a greater prevalence of smoking and alcohol drinking (all *P* < 0.001). In contrast, shift work, chemical substance exposure, noise exposure, and dust exposure did not differ significantly by tea consumption category. Clusters of cardiometabolic conditions—including hypertension, diabetes, hyperlipidemia, and cardiovascular disease—also varied across groups, with the highest prevalence observed among high-level tea drinkers (all *P* < 0.05). These patterns indicate that tea consumption in this population is closely associated with multiple lifestyle and health-related characteristics.

**TABLE 1:** Baseline characteristics of participants.

Variables	Tea consumption levels				*P*-value
	Total (*N* = 2,574)	None (*N* = 1,276)	Low (*N* = 679)	High (*N* = 619)	
Age, year	41.05 (8.21)	39.50 (8.15)	41.67 (7.96)	43.54 (7.92)	<0.001
Sex					<0.001
Male	1,701 (66.08%)	678 (53.13%)	495 (72.90%)	528 (85.30%)	–
Female	873 (33.92%)	598 (46.87%)	184 (27.10%)	91 (14.70%)	–
Ethnicity					0.676
Han	2,526 (98.14%)	1,253 (98.20%)	668 (98.38%)	605 (97.74%)	–
Other	48 (1.86%)	23 (1.80%)	11 (1.62%)	14 (2.26%)	–
Marital status					0.055
Married	2,440 (96.71%)	1,227 (97.15%)	625 (95.27%)	588 (97.35%)	–
Unmarried/separated	83 (3.29%)	36 (2.85%)	31 (4.73%)	16 (2.65%)	–
Shift work					0.586
No	804 (31.24%)	396 (31.03%)	205 (30.19%)	203 (32.79%)	–
Yes	1,770 (68.76%)	880 (68.97%)	474 (69.81%)	416 (67.21%)	–
Chemical substance exposure					0.163
No	596 (23.15%)	290 (22.73%)	174 (25.63%)	132 (21.32%)	–
Yes	1,978 (76.85%)	986 (77.27%)	505 (74.37%)	487 (78.68%)	–
Noise exposure					0.303
No	973 (37.80%)	495 (38.79%)	240 (35.35%)	238 (38.45%)	–
Yes	1,601 (62.20%)	781 (61.21%)	439 (64.65%)	381 (61.55%)	–
Dust exposure					0.066
No	1,918 (74.51%)	971 (76.10%)	484 (71.28%)	463 (74.80%)	–
Yes	656 (25.49%)	305 (23.90%)	195 (28.72%)	156 (25.20%)	–
Cigarette smoking					<0.001
No	1,373 (53.34%)	868 (68.03%)	318 (46.83%)	187 (30.21%)	–
Yes	1201 (46.66%)	408 (31.97%)	361 (53.17%)	432 (69.79%)	–
Alcohol drinking					<0.001
No	1,605 (62.35%)	947 (74.22%)	368 (54.20%)	290 (46.85%)	–
Yes	969 (37.65%)	329 (25.78%)	311 (45.80%)	329 (53.15%)	–
Physical activity					<0.001
Inactive	616 (23.93%)	344 (26.96%)	124 (18.26%)	148 (23.91%)	–
Active	1,958 (76.07%)	932 (73.04%)	555 (81.74%)	471 (76.09%)	–
Hypertension					<0.001
No	1,972 (76.61%)	1,030 (80.72%)	521 (76.73%)	421 (68.01%)	–
Yes	602 (23.39%)	246 (19.28%)	158 (23.27%)	198 (31.99%)	–
Diabetes					<0.001
No	2,378 (92.39%)	1,206 (94.51%)	619 (91.16%)	553 (89.34%)	–
Yes	196 (7.61%)	70 (5.49%)	60 (8.84%)	66 (10.66%)	–
Hyperlipidemia					<0.001
No	1,494 (58.04%)	826 (64.73%)	377 (55.52%)	291 (47.01%)	–
Yes	1,080 (41.96%)	450 (35.27%)	302 (44.48%)	328 (52.99%)	–
Cardiovascular disease					0.046
No	2,383 (92.58%)	1,195 (93.65%)	628 (92.49%)	560 (90.47%)	–
Yes	191 (7.42%)	81 (6.35%)	51 (7.51%)	59 (9.53%)	–

### 3.2 Association between tea consumption and skeletal muscle mass

Table 2 summarizes the associations between tea consumption and skeletal muscle mass in the overall sample and across SES strata. In the unadjusted model (Model 1), both low- and high-level tea drinking showed strong positive associations with skeletal muscle mass compared with non-tea drinkers. These associations were attenuated but remained statistically significant after adjusting for basic demographic factors (Model 2). In the fully adjusted model (Model 3), which accounted for occupational exposures, lifestyle behaviors, and cardiometabolic conditions, tea consumption continued to demonstrate an independent positive association with muscle mass. In the total population, low-level tea drinking was associated with a 0.105-unit increase in skeletal muscle mass (95% CI: 0.042–0.167, *P* = 0.001), and high-level consumption was associated with a 0.137-unit increase (95% CI: 0.070–0.205, *P* < 0.001), relative to non-drinkers.

**TABLE 2:** Association between tea consumption and skeletal muscle mass among oilfield workers.

Tea consumption level	Model 1		Model 2		Model 3	
	β (95% CI)	*P*-value	β (95% CI)	*P*-value	β (95% CI)	*P*-value
Total population						
Non-tea drinking	Reference	–	Reference	–	Reference	–
Low-level tea drinking	0.459 (0.351, 0.567)	<0.001	0.097 (0.029, 0.164)	0.005	0.105 (0.042, 0.167)	0.001
High-level tea drinking	0.748 (0.637, 0.859)	<0.001	0.167 (0.095, 0.239)	<0.001	0.137 (0.070, 0.205)	<0.001
Low SES						
Non-tea drinking	Reference	–	Reference	–	Reference	–
Low-level tea drinking	0.447 (0.284, 0.610)	<0.001	0.058 (–0.040, 0.155)	0.246	0.069 (−0.022, 0.160)	0.135
High-level tea drinking	0.841 (0.674, 1.009)	<0.001	0.186 (0.082, 0.290)	<0.001	0.172 (0.075, 0.270)	0.001
Medium SES						
Non-tea drinking	Reference	–	Reference	–	Reference	–
Low-level tea drinking	0.407 (0.188, 0.627)	<0.001	0.009 (−0.136, 0.154)	0.904	0.058 (−0.078, 0.194)	0.401
High-level tea drinking	0.766 (0.545, 0.988)	<0.001	0.139 (−0.012, 0.290)	0.072	0.111 (−0.031, 0.253)	0.127
High SES						
Non-tea drinking	Reference	–	Reference	–	Reference	–
Low-level tea drinking	0.552 (0.368, 0.736)	<0.001	0.211 (0.093, 0.328)	<0.001	0.182 (0.073, 0.291)	0.001
High-level tea drinking	0.658 (0.462, 0.854)	<0.001	0.160 (0.030, 0.289)	0.016	0.132 (0.010, 0.253)	0.034

Model 1, no covariate was adjusted. Model 2, adjusted for age, sex, ethnicity, marital status, and socioeconomic status. Model 3, further adjusted for shift work, chemical substance exposure, noise exposure, dust exposure, cigarette smoking, alcohol drinking, physical activity, hypertension, diabetes, hyperlipidemia, and cardiovascular disease. Socioeconomic status stratification analysis does not adjust for socioeconomic status. SES, socioeconomic status; CI, confidence interval.

SES-stratified analyses revealed heterogeneous associations. Among individuals with low SES, only high-level tea consumption remained significantly associated with higher muscle mass in Model 3 (β = 0.172, 95% CI: 0.075–0.270, *P* = 0.001). In the medium SES group, neither low- nor high-level tea intake showed statistically significant associations after full adjustment. By contrast, among participants with high SES, both low-level (β = 0.182, 95% CI: 0.073–0.291, *P* = 0.001) and high-level tea drinking (β = 0.132, 95% CI: 0.010–0.253, *P* = 0.034) remained positively associated with muscle mass. Taken together, these findings indicate that habitual tea consumption is linked to greater skeletal muscle mass, but the strength and significance of this association vary across socioeconomic strata.

### 3.3 Interaction analyses

To further examine whether the association between tea consumption and skeletal muscle mass differed by socioeconomic status, we fitted a fully adjusted interaction model including the cross-product terms between tea consumption categories and SES levels. The interaction effects demonstrated a heterogeneous pattern across SES strata (Supplementary Table 2).

No statistically significant interaction was observed between tea consumption and medium SES for either low-level or high-level tea drinking, indicating that the association between tea intake and muscle mass did not differ meaningfully between medium-SES workers and the low-SES reference group. Similarly, the interaction terms for high-level tea drinking in the high SES group were not significant (*P* = 0.978), suggesting no detectable modification at higher intake levels. In contrast, the interaction term for low-level tea consumption among participants in the high SES group reached statistical significance (β = 0.156, *P* = 0.031). This finding indicates that individuals with high SES experience a greater increase in skeletal muscle mass from low-level tea consumption compared with those in the lowest SES group. Taken together, the results point to a modest but noteworthy SES-related heterogeneity, with the beneficial association of tea intake appearing more pronounced for low-level consumption among workers in the highest SES category.

### 3.4 Smooth curve fitting analyses

**FIGURE 2:**
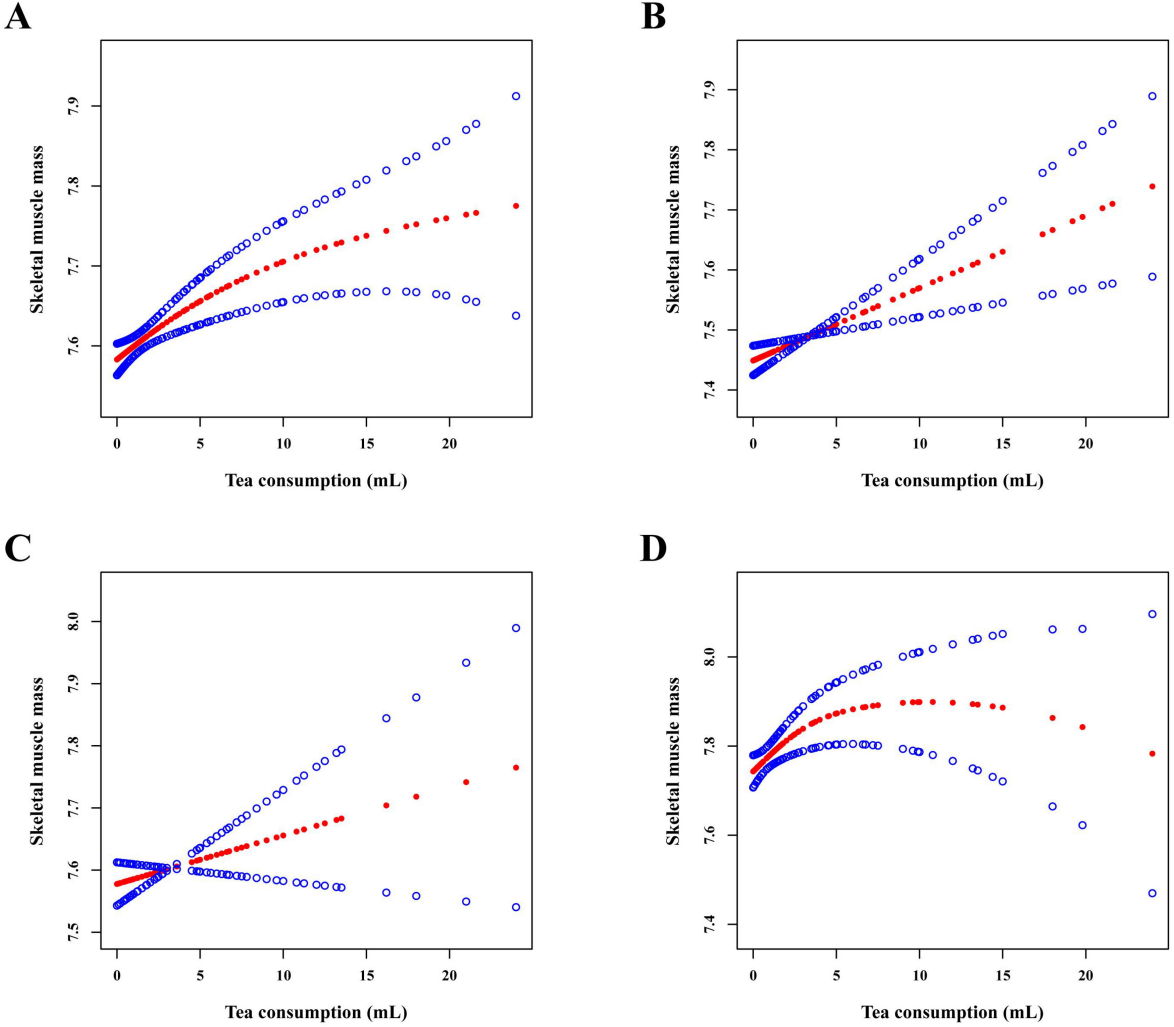
The smooth curve fitting analysis between tea consumption and skeletal muscle mass among oilfield workers. **(A)** Total population. **(B)** Low socioeconomic status. **(C)** Medium socioeconomic status. **(D)** High socioeconomic status. The red line represents the smooth curve fit between variables. The blue line represents the 95% confidence interval of the fit. Tea consumption was divided by 500 prior to analysis to improve model convergence and interpretability. Age, sex, ethnicity, marital status, socioeconomic status, shift work, chemical substance exposure, noise exposure, dust exposure, cigarette smoking, alcohol drinking, physical activity, hypertension, diabetes, hyperlipidemia, and cardiovascular disease were adjusted. Socioeconomic status stratification analysis does not adjust for socioeconomic status.

Smooth curve fitting analyses were further employed to illustrate dose–response associations between tea consumption and skeletal muscle mass (Figure 2). In the total population, skeletal muscle mass increased steadily with higher levels of tea consumption, indicating an approximately linear positive association. Stratified analyses revealed heterogeneity across socioeconomic groups. Among individuals with low SES, the association remained generally linear and upward, suggesting consistent benefits with increasing tea intake. In the medium SES group, the curve was relatively flat, indicating a non-significant relationship. In contrast, the high SES group exhibited a clearly non-linear pattern resembling an inverted U-shape, with muscle mass rising at lower to moderate levels of tea consumption and then leveling off or declining slightly at higher intakes, suggesting a potential threshold beyond which additional tea intake conferred no further advantage. These patterns underscore SES-related differences in the dose-response shape.

### 3.5 Sensitivity analyses

The findings remained robust across a series of sensitivity analyses. First, when tea consumption was reclassified as a binary exposure, the direction and magnitude of the associations with skeletal muscle mass were largely unchanged, indicating that the results were not dependent on the specific categorization of tea intake (Supplementary Table 3). Second, analyses based on multiple imputation for missing covariates produced estimates that closely aligned with those from the primary models, further supporting the stability of the observed associations (Supplementary Table 4). Across all sensitivity analyses, the positive relationship between tea consumption and skeletal muscle mass persisted. These results reinforce the reliability of the main findings and suggest that they are not attributable to data handling procedures or model specification.

## 4 Discussion

This study examined the association between tea consumption and skeletal muscle mass among oilfield workers and explored how this relationship varied across SES levels. We observed that tea consumption was positively associated with muscle mass in individuals with low SES, while no significant association was found among those with middle SES. Interestingly, the smooth curve analysis indicated a potential inverted U-shaped pattern in the high SES group, suggesting that moderate tea intake may be most favorable for muscle health, whereas higher levels offer no additional benefit. These findings highlight the importance of considering socioeconomic context when evaluating the health impacts of dietary behaviors.

Our results add to a growing body of evidence linking dietary factors to muscle health. Previous studies have suggested that tea contains multiple bioactive components with antioxidant and anti-inflammatory properties relevant to muscle metabolism (23, 24). However, potential threshold effects at higher intake levels have also been noted (25). Beyond general antioxidant effects, several bioactive compounds in tea may plausibly contribute to skeletal muscle preservation. Tea is rich in polyphenols, particularly catechins such as epigallocatechin gallate, which have been shown to modulate oxidative stress and inflammatory signaling pathways closely involved in muscle protein turnover (13, 14). Experimental studies suggest that tea polyphenols may attenuate muscle atrophy by reducing reactive oxygen species accumulation, improving mitochondrial function, and suppressing chronic low-grade inflammation—processes that are central to age- and stress-related muscle loss (13, 26). In addition, L-theanine, a tea-specific amino acid, has been reported to influence neuroendocrine stress responses and immune regulation (15). Given that chronic psychosocial and occupational stress can disrupt muscle metabolism via hormonal and inflammatory pathways, L-theanine may indirectly support muscle maintenance, particularly in populations exposed to sustained physical or environmental stress. Although the bioavailability and effective dose of these compounds vary by tea type and preparation, habitual tea consumption may represent a meaningful population-level source of these bioactives that act cumulatively over time.

From a social epidemiological perspective, the finding that tea consumption was more beneficial in the low SES group provides valuable insight into health inequality. Individuals with lower socioeconomic position often face greater psychological stress, reduced access to health-promoting resources, and more constrained dietary choices, which can raise baseline risk and create more room for benefit from relatively simple interventions (27–29). Empirical evidence indicates that the mortality risk reductions associated with physical activity are often larger among socioeconomically disadvantaged groups (8). Conversely, large cohort analyses have reported that the apparent protective association of light-to-moderate alcohol consumption with ischemic heart disease mortality is more pronounced in higher SES groups (9). However, evidence regarding smoking is somewhat different in nature, as disadvantaged smokers generally exhibit lower quit rates and encounter greater barriers that limit their ability to fully benefit from cessation (7). Together, these examples illustrate how the adoption, effectiveness, and net health returns of health-related behaviors can vary by socioeconomic context—a framing that helps explain why tea consumption might yield differential muscle-health benefits across SES strata.

Educational attainment, as a core component of the SES index used in this study, may play a particularly important role in shaping the observed heterogeneity. Education reflects cumulative socioeconomic advantage across the life course and is closely linked to health literacy, dietary knowledge, and the capacity to adopt and sustain health-promoting behaviors. In occupational populations, educational level may also influence job roles, work autonomy, and exposure intensity, which in turn affect nutritional status and physiological resilience. In addition, annual income captures current material resources that may further condition the health effects of tea consumption. Higher income can facilitate access to higher-quality diets, adequate protein intake, and supportive living conditions, which may enhance the biological effectiveness of tea-related bioactive compounds. Conversely, limited economic resources may constrain the extent to which a single dietary behavior translates into measurable physiological gains. Taken together, the combination of education and income allows the SES index to reflect both long-term social advantage and contemporaneous material conditions, providing a more comprehensive framework for interpreting the socioeconomic patterning observed in the tea–muscle mass association.

The occupational context of oilfield workers further underscores the relevance of our findings. This population is typically exposed to chemical, noise, and dust hazards, which can contribute to oxidative stress and inflammation—pathways that are also implicated in sarcopenia and metabolic dysfunction (30, 31). Tea consumption may partially counteract these adverse effects through antioxidative mechanisms, particularly in workers with fewer socioeconomic resources. This suggests that promoting accessible and culturally acceptable dietary behaviors like tea drinking could serve as a low-cost, population-specific strategy to mitigate occupational health disparities.

Several mechanisms may underlie the observed SES-specific associations. First, differences in nutritional status, dietary diversity, and overall health behaviors across SES groups could modify the bioavailability and metabolic effects of tea constituents (28). Second, psychosocial stress and sleep patterns—both closely tied to socioeconomic disadvantage—may influence oxidative balance and muscle metabolism, potentially amplifying the benefits of tea’s antioxidant properties in disadvantaged groups (27, 29). Finally, the non-linearity observed in the high SES group could reflect higher caffeine or fluoride intake from excessive tea consumption, which has been associated with altered mineral balance and metabolic strain (21, 25). Taken together, these patterns suggest that tea consumption may operate as a complementary behavior that reinforces existing advantages among higher SES individuals, whereas for those with socioeconomic disadvantages, tea may primarily serve as a compensatory behavior, mitigating nutritional or oxidative deficits. This dual pathway—amplification at the top and compensation at the bottom—offers a coherent explanation for the SES-differentiated associations identified in our study. Beyond antioxidant activity, accumulating evidence suggests that tea polyphenols may influence skeletal muscle metabolism through anti-inflammatory and immunomodulatory pathways, including suppression of low-grade systemic inflammation and modulation of cytokine signaling involved in muscle protein turnover (26, 32, 33). Tea constituents may also interact synergistically with dietary protein and micronutrients, enhancing anabolic signaling and mitochondrial efficiency (34). Moreover, occupational exposures common among oilfield workers—such as chemical agents and particulate matter—can exacerbate oxidative stress and inflammation, potentially modifying the physiological efficacy of tea bioactives (35). These interacting pathways provide a plausible biological framework through which socioeconomic context may shape the magnitude of tea-related muscle health benefits.

From a policy perspective, our findings highlight the need to tailor diet-based prevention strategies to socioeconomic realities. Although tea drinking is culturally embedded and widely accessible, its benefits were not evenly distributed. Structural disadvantages may limit individuals’ capacity to convert healthy behaviors into measurable physiological gains. Occupational health programs—especially in resource-limited industrial settings—should therefore incorporate supportive measures such as improving workplace nutrition environments, reducing physical workload strain, and promoting equitable access to preventive health services. Such upstream actions may create conditions in which simple behaviors like tea consumption can yield meaningful improvements in muscle health and reduce SES-related disparities.

The present study has several strengths. It is among the first to investigate socioeconomic disparities in the relationship between tea consumption and muscle mass within an occupational population. The use of a validated equation to estimate ASM and the inclusion of comprehensive lifestyle and occupational covariates strengthen the internal validity of our findings. Additionally, multiple sensitivity analyses confirmed the robustness of the results. However, several limitations should be considered when interpreting these findings. First, the cross-sectional nature of the analysis limits the ability to establish temporal or causal relationships; thus, the associations observed may reflect correlation rather than true causation. Second, although muscle mass was estimated using validated prediction formulas, rather than quantified through gold-standard techniques such as DXA, some degree of measurement imprecision is inevitable. Third, information on tea consumption was derived from self-reported questionnaires, which are subject to recall inaccuracies and reporting bias. Detailed information on tea type, brewing strength, preparation methods, and concurrent intake of other functional beverages was not available. Such factors may influence the bioavailability of tea polyphenols and should be incorporated into future studies to reduce exposure misclassification. Fourth, despite adjusting for a wide range of sociodemographic, behavioral, and health-related variables, unmeasured or inadequately measured confounders—such as overall dietary quality, genetic predisposition—may still influence the observed relationships. Furthermore, although inflammation represents a biologically plausible mediator of the tea-muscle mass association, inflammatory biomarkers such as C-reactive protein or interleukin-6 were not available for mediation analysis in the present dataset. Future studies incorporating biomarker data are needed to formally test inflammatory pathways. Fifth, while the relative homogeneity of occupational roles and insurance coverage reduces residual confounding, future studies incorporating more comprehensive SES indicators may further refine the assessment of social stratification in diet–muscle health associations. Finally, the study population consisted of oilfield workers, a relatively specialized occupational group, which may limit the extrapolation of our findings to broader populations. Future longitudinal and mechanistic studies are needed to validate these associations and clarify the underlying pathways.

## 5 Conclusion

In conclusion, our findings reveal clear socioeconomic disparities in the association between tea consumption and skeletal muscle mass among oilfield workers. Tea consumption was positively associated with muscle mass in the overall population. SES-stratified analyses further showed that this association remained significant for both low- and high-level tea intake in the high SES group, while only high-level intake was significant among individuals with low SES, and no significant associations were observed in the medium SES group. These results highlight the socioeconomic patterning of diet-related muscle health and underscore the need to incorporate social context into prevention strategies. Future research should build on these findings through randomized controlled trials evaluating the effects of tea supplementation on muscle synthesis and function, particularly in socioeconomically disadvantaged populations. Integrative multi-omics approaches—such as metabolomics and gut microbiome profiling—may help elucidate molecular and microbial mediators of tea’s bioactivity. Longitudinal studies are also warranted to examine how socioeconomic conditions shape long-term trajectories of tea consumption and muscle health across the life course.

## Data Availability

The raw data supporting the conclusions of this article will be made available by the authors, without undue reservation.

## Footnotes

1
http://www.empowerstats.com
