# Supplementary material for: Socioeconomic stratification in the association between tea consumption and skeletal muscle mass among oilfield workers
Source: Front Nutr. 2026 Apr 24;13:1777775. doi: 10.3389/fnut.2026.1777775 (PMC13152726; doi:10.3389/fnut.2026.1777775)
Supplement: Supplementary file 1 [file Data_Sheet_1.pdf]

Supplementary Material

Table S1. Category and definition of covariates.

Table S2. Interaction effects of tea consumption and socioeconomic status on skeletal muscle mass index.

Table S3. Association between tea consumption and skeletal muscle mass among oilfield workers, using binary tea drinking status.

Table S4. Association between tea consumption and skeletal muscle mass among oilfield workers, after employing imputation for covariates.

Table S1. Category and definition of covariates.

Variable	Category and definition
Shift work	Shift work is defined as the regular rotation of individuals to work outside the hours of 8:00 AM to 5:00 PM for a minimum duration of one year.
Chemical substance exposure	Chemical substance exposure is defined as the self-reported exposure of individuals to hazardous chemical substances in the workplace or environment, including benzene, toluene, xylene, hydrogen sulfide, carbon monoxide, nitrogen oxides, carbon tetrachloride, n-hexane, n-pentane, gasoline, etc.
Noise exposure	Noise exposure is defined as the self-reported exposure of individuals to noise levels in the work environment that exceed legal noise standards.
Dust exposure	Dust exposure is defined as the self-reported exposure of individuals to inhalable or respirable dust particles during the work process, which may originate from production processes, material handling, or the surrounding environment.
Cigarette smoking	Cigarette smoking is defined as individuals who smoke at least one cigarette daily for six months or longer.
Alcohol drinking	Alcohol drinking is defined as those who consume alcohol at least once a week and maintain this frequency for six months or longer.
Physical activity	Physical activity is assessed based on questionnaire items asking participants whether they usually engaged in moderate-intensity activities—such as slow jogging or moderate-paced cycling—lasting at least 10 minutes per session.
Hypertension	Hypertension is defined as systolic blood pressure ≥140 mmHg, diastolic blood pressure ≥90 mmHg, self-reported physician diagnosis of hypertension, or current use of antihypertensive medication.
Diabetes	Diabetes is defined as fasting plasma glucose ≥7.0 mmol/L, glycated hemoglobin ≥6.5%, self-reported physician diagnosis of diabetes, or current use of glucose-lowering medication.
Hyperlipidemia	Hyperlipidemia is defined as total cholesterol ≥6.2 mmol/L, triglycerides ≥2.3 mmol/L, low-density lipoprotein cholesterol ≥4.1 mmol/L, high-density lipoprotein cholesterol <1.0 mmol/L, self-reported physician diagnosis of hyperlipidemia, or current use of lipid-lowering medication.
Cardiovascular disease	Cardiovascular disease is defined as a self-reported history of coronary heart disease, atherosclerosis, or stroke.

Table S2. Interaction effects of tea consumption and socioeconomic status on skeletal muscle mass index.

Variables	*β* (interaction term)	SE	95% CI	*P* value
Low-level tea drinking × medium SES	0.006	0.077	-0.146 to 0.157	0.943
High-level tea drinking × medium SES	-0.023	0.078	-0.176 to 0.131	0.774
Low-level tea drinking × high SES	0.156	0.072	0.014 to 0.298	0.031
High-level tea drinking × high SES	0.002	0.076	-0.147 to 0.151	0.978

The model was adjusted for age, sex, ethnicity, marital status, shift work, chemical substance exposure, noise exposure, dust exposure, cigarette smoking, alcohol consumption, physical activity, hypertension, diabetes, hyperlipidemia, and cardiovascular disease.

Table S3. Association between tea consumption and skeletal muscle mass among oilfield workers, using binary tea drinking status.

Tea consumption level	Model 1		Model 2		Model 3	
	β **(95% CI)**	***P*** **value**	***β*** **(95% CI)**	***P*** **value**	***β*** **(95% CI)**	***P*** **value**
**Total population**						
Non-tea drinking	Reference		Reference		Reference	
Tea drinking	0.597 (0.507, 0.687)	<0.001	0.128 (0.07, 0.186)	<0.001	0.119 (0.065, 0.173)	<0.001
**Low SES**						
Non-tea drinking	Reference		Reference		Reference	
Tea drinking	0.637 (0.499, 0.774)	<0.001	0.115 (0.031, 0.199)	0.007	0.116 (0.037, 0.195)	0.004
**Medium SES**						
Non-tea drinking	Reference		Reference		Reference	
Tea drinking	0.585 (0.403, 0.766)	<0.001	0.069 (-0.054, 0.193)	0.272	0.083 (-0.034, 0.199)	0.166
**High SES**						
Non-tea drinking	Reference		Reference		Reference	
Tea drinking	0.6 (0.447, 0.754)	<0.001	0.189 (0.088, 0.29)	<0.001	0.161 (0.066, 0.255)	0.001

Model 1, no covariate was adjusted. Model 2, adjusted for age, sex, ethnicity, marital status, and socioeconomic status. Model 3, further adjusted for shift work, chemical substance exposure, noise exposure, dust exposure, cigarette smoking, alcohol drinking, physical activity, hypertension, diabetes, hyperlipidemia, and cardiovascular disease. Socioeconomic status stratification analysis does not adjust for socioeconomic status. SES, socioeconomic status; CI, confidence interval.

Table S4. Association between tea consumption and skeletal muscle mass among oilfield workers, after employing imputation for covariates.

Tea consumption level	Model 1		Model 2		Model 3	
	β **(95% CI)**	***P*** **value**	***β*** **(95% CI)**	***P*** **value**	***β*** **(95% CI)**	***P*** **value**
**Total population**						
Non-tea drinking	Reference		Reference		Reference	
Low-level tea drinking	0.443 (0.343, 0.543)	<0.001	0.107 (0.045, 0.169)	0.001	0.106 (0.048, 0.164)	<0.001
High-level tea drinking	0.740 (0.637, 0.843)	<0.001	0.177 (0.111, 0.244)	<0.001	0.138 (0.076, 0.200)	<0.001
**Low SES**						
Non-tea drinking	Reference		Reference		Reference	
Low-level tea drinking	0.453 (0.304, 0.603)	<0.001	0.078 (-0.012, 0.167)	0.091	0.076 (-0.008, 0.161)	0.077
High-level tea drinking	0.828 (0.675, 0.980)	<0.001	0.183 (0.087, 0.278)	<0.001	0.153 (0.063, 0.243)	0.001
**Medium SES**						
Non-tea drinking	Reference		Reference		Reference	
Low-level tea drinking	0.432 (0.226, 0.639)	<0.001	0.017 (-0.118, 0.152)	0.801	0.058 (-0.070, 0.186)	0.377
High-level tea drinking	0.783 (0.577, 0.989)	<0.001	0.152 (0.012, 0.293)	0.034	0.110 (-0.023, 0.243)	0.106
**High SES**						
Non-tea drinking	Reference		Reference		Reference	
Low-level tea drinking	0.476 (0.303, 0.649)	<0.001	0.210 (0.101, 0.318)	<0.001	0.179 (0.078, 0.280)	0.001
High-level tea drinking	0.634 (0.451, 0.818)	<0.001	0.183 (0.064, 0.303)	0.003	0.151 (0.039, 0.263)	0.008

Model 1, no covariate was adjusted. Model 2, adjusted for age, sex, ethnicity, marital status, and socioeconomic status. Model 3, further adjusted for shift work, chemical substance exposure, noise exposure, dust exposure, cigarette smoking, alcohol drinking, physical activity, hypertension, diabetes, hyperlipidemia, and cardiovascular disease. Socioeconomic status stratification analysis does not adjust for socioeconomic status. SES, socioeconomic status; CI, confidence interval.
